# An information theory-based approach to characterize drivers of upstream salmon migration

**DOI:** 10.1371/journal.pone.0269193

**Published:** 2022-06-09

**Authors:** Allison Goodwell, Nicholas Campbell

**Affiliations:** Department of Civil Engineering, University of Colorado Denver, Denver, CO, United States America; Texas A&M University, UNITED STATES

## Abstract

The migration timing of Pacific salmon in the Columbia River basin is subject to multiple influences related to climate, human water resource management, and lagged effects such as oceanic conditions. We apply an information theory-based approach to analyze drivers of adult Chinook salmon migration within the spring and fall spawning seasons and between years based on salmon counts at dams along the Columbia and Snake Rivers. Time-lagged mutual information and information decomposition measures, which characterize lagged and nonlinear dependencies as reductions in uncertainty, are used to detect interactions between salmon counts and lagged streamflows, air and water temperatures, precipitation, snowpack, climate indices and downstream salmon counts. At a daily timescale, these interdependencies reflect migration timing and show differences between fall and spring run salmon, while dependencies based on variables at an annual resolution reflect long-term predictability. We also highlight several types of joint dependencies where predictability of salmon counts depends on the knowledge of multiple lagged sources. This study illustrates how co-varying human and natural drivers could propagate to influence salmon migration timing or overall returns, and how nonlinear types of dependencies between variables enhance predictability of a target. This information-based framework is broadly applicable to assess driving factors in other types of complex water resources systems or species life cycles.

## 1 Introduction

Salmon population abundances have declined from historical estimates in the Pacific Northwest, with the Chinook Salmon (Oncorhynchus tshawytscha) species listed as endangered or threatened for all segments of the Columbia and Snake rivers [1]. Like other anadromous fish, most Pacific salmon home, or return, to their freshwater birth location when it is time to spawn, after living several years in the ocean [2], and migration timing during the freshwater stages is a predictor of survival in other life stages [3]. Hydroelectric dams play a central role in salmon migration within the Columbia River Basin, as they alter natural flows, water temperatures, and dissolved gas levels that influence salmon health, migration timing, and habitat [4]. For example, when water temperature increases to around 70°F, salmon face health risks that compound the stresses inherent to migration [5]. Additionally, all salmon must pass through each dam along the river twice during their lifetimes, which presents a compounding effect for salmon that originate from farther upstream [6]. Efforts to directly increase salmon populations include “trap-and-haul”, or the transportation of juveniles downstream or adults upstream [7, 8], but it is often difficult to assess the efficacy of these operations [8]. Other mitigation efforts in the Columbia River Basin include fish ladders and juvenile bypass systems at dams [9] and coordinated spilling procedures and flow augmentation from higher elevation, colder reservoirs [10, 11]. These efforts have varying influences on salmon migration dynamics. For example, high temperatures at fish ladders have been found to cause migration delays [12], and managed pulse flows do not always lead to significant changes in migration [13]. Meanwhile, dams provide renewable energy and other ecosystem and economic services, such that salmon populations are only one aspect of a complex system spanning multiple and often conflicting objectives [14, 15] with economic, environmental, or cultural importance.

In general, both human and natural drivers influence salmon migratory dynamics and population abundances. These factors, such as stream flows, reservoir storage and spill, water temperature, water quality, ocean conditions, and climate dynamics also interact with each other, resulting in complex and nonlinear interdependencies. These interdependencies also combine with physiological drivers of salmon. Previous studies highlight the importance of considering salmon dynamics as a complex network, where multivariate interactions occur with different timescales and strengths. For example, considering nonlinear environmental drivers has been found to improve forecast accuracy in fisheries models [16] and the extent to which a variable is predictive can depend on nonlinear thresholds [13]. It has also been found that there are multiple relevant indicators of salmon returns [17], carryover effects within salmon lifecyles in which conditions experienced at one life stage affect a subsequent stage [18] and marine survival [19], but no single indicator or subset of indicators is highly explanatory. Moreover, it has been found that the relationships between indicator variables themselves can be non-stationary [20, 21], which has implications for predictive skills of models based on certain input combinations, and indicates the difficulty in distinguishing causal drivers from temporary correlations. Within this complex system, the particular influence of an individual driver remains uncertain [22], and the system can be considered non-interventional in that we cannot “intervene” or perturb the dynamics and isolate a response [23]. This necessitates the inference of casual dependencies using statistical methods, and the differentiation between correlations and causality.

**Fig 1:**
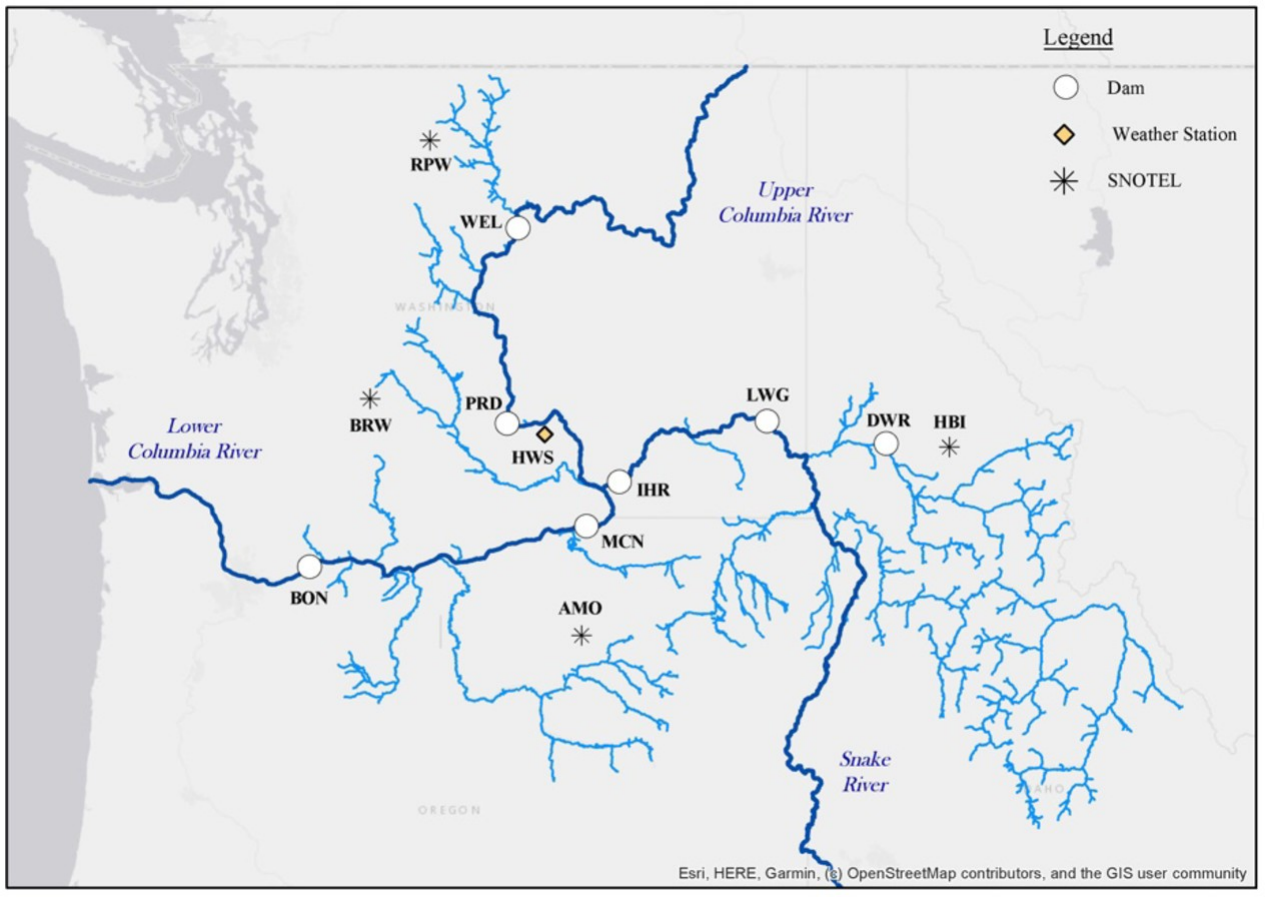
Map of Columbia River Basin with dams and observation sites marked. Abbreviations are listed in Table 1. Flow and temperature gage stations correspond with dam locations (white circles). SNOTEL sites correspond with snow-water-equivalent (SWE) measurements.

We focus on drivers of salmon migration as an illustrative case where information theory-based metrics, which measure information flows as reductions in uncertainty, provide a useful framework to study a complex system with many interdependent aspects. We particularly use video count data to focus on drivers of upstream migration timing of adult Chinook salmon on a daily timescale, which is relevant in terms of seasonal migration dynamics, and adult population abundances at dams on a multi-year timescale, which reflects the entire salmon life-cycle and long term effects on salmon. Although numerous species of salmon and steelhead inhabit this region, Chinook are an important species in the two distinct ecosystems of the Columbia River Basin and Pacific Ocean [24]. From daily data, we study influences on migration timing between locations, and from annual total counts, we consider lagged predictors of population abundances that pass different sites from year to year. We take a complex network perspective [25] on salmon migration dynamics between dams as measured by video-based counts. Specifically, we consider temporal dynamics overlaid on a spatial network formed by dams along the Columbia and Snake Rivers, where time-dependent couplings between counts and environmental variables reveal individual and joint types of interactions between them. We use an information theory-based framework to characterize a “process network” [26] of lagged dependencies between daily and annual salmon counts, temperatures, streamflow rates, and other variables at dams along the Upper Columbia, Snake, and Lower Columbia reaches (Fig 1, Table 1).

**Table 1:** Time-series variables included in analyses of CRB salmon counts. Chinook counts were obtained from the Fish Passage Center [39]. Water temperatures and flow rates were obtained from USACE and USGS [41, 45]. Air temperature and precipitation were obtained from the Hanford Site [42]. Snow-water-equivalent (SWE) was obtained from NRCS [44]. The PDO was obtained from NOAA [46].

Variables**	Variable Abbr.	Site Abbr.	Location	Analysis type*
Chinook counts at Bonneville Dam	BON	BON	45.644, -121.940	10 yr, 35 yr
Chinook counts at McNary Dam	MCN	MCN	45.936, -119.298	10 yr, 35 yr
Chinook counts at Priest Rapids Dam	PRD	PRD	46.644, -119.910	10 yr, 35 yr
Chinook counts at Wells Dam	WEL	WEL	47.948, -119.865	10 yr, 35 yr
Chinook counts at Ice Harbor Dam	IHR	IHR	46.250, -118.879	10 yr, 35 yr
Chinook counts at Lower Granite Dam	LWG	LWG	46.661, -117.428	10 yr, 35 yr
Flow at Bonneville Dam	Q_BON_	BON	45.644, -121.940	10 yr, 35 yr
Flow at Lower Granite Dam	Q_LWG_	LWG	46.661, -117.428	10 yr, 35 yr
Flow at Priest Rapids Dam	Q_PRD_	PRD	46.644, -119.910	10 yr, 35 yr
Water temperature at BON	T_BON_	BON	45.644, -121.940	10 yr, 35 yr
Water temperature at LWG	T_LWG_	LWG	46.661, -117.428	10 yr, 35 yr
Water temperature at PRD	T_PRD_	PRD	46.644, -119.910	10 yr, 35 yr
Flow at Dworshak Dam	Q_DWR_	DWR	46.515, -116.298	35 yr
**Spill at Dworshak Dam**	Sp_DWR_	DWR	46.515, -116.298	35 yr
Days with water temp > 68°F at BON	TD_BON68_	BON	45.644, -121.940	35 yr
Days with water temp > 70°F at BON	TD_BON70_	BON	45.644, -121.940	35 yr
**Days with water temp >68**°F at PRD	TD_PRD68_	PRD	46.644, -119.910	35 yr
Days with water temp > 70°F at PRD	TD_PRD70_	PRD	46.644, -119.910	35 yr
**Days with water temp >68**°F at LWG	TD_LWG68_	LWG	46.661, -117.428	35 yr
Days with water temp> 70°F at LWG	TD_LWG70_	LWG	46.661, -117.428	35 yr
**Precipitation at The Hanford Site**	Precip	HWS	46.557, -119.526	35 yr
**Air temperature at the Hanford Site**	Ta	HWS	46.557, -119.526	35 yr
**SWE at Rainy Pass, WA**	SWE_UpCRB_	RPW	48.583, -120.718	35 yr
**SWE at Bumping Ridge, WA**	SWE_MidCRB_	BRW	46.817, -121.333	35 yr
SWE at Arbuckle Mountain, OR	SWE_LowCRB_	AMO	45.19, -119.25	35 yr
**SWE at Hemlock Butte, ID**	SWE_Snake_	HBI	46.483, -115.63	35 yr
**Pacific Decadal Oscillation Index**	PDO	N/A	N/A	35 yr

*35-year (annual totals), 10-year (daily)

**bold variables: MI statistically significant in 35-yr analysis for counts for at least one site (excluding counts themselves)

Process networks have previously been constructed to analyze the influence of weather conditions on joint variability of atmospheric states [27–29], connectivity between ecosystem fluxes [26, 30], biosphere responses to climate forcing [31], and spatial and temporal connectivity within a delta system [32]. Information theory-based measures have also been used in a water resources management context to infer drivers behind reservoir release decisions [33, 34] and connectivity between precipitation and streamflow [35]. Advantages of an information theory framework include an inherent focus on predictability and uncertainty, as measures quantify reductions in uncertainty, and the detection of both linear and non-linear types of dependencies that can range from pairwise to highly multivariate [36]. In general, process networks enable us to address questions pertaining to causality and predictability [37, 38]. This is the first application of process networks to a biological species, and can be used to identify combinations of dominant drivers and risk factors within a migratory network. Here, questions include “given the knowledge of adult salmon returning from the ocean, how impactful are flow rates and temperatures to salmon counts at upstream dams?” and “how interdependent are salmon counts between neighboring and distant dams?” In addition to quantifying the timescales associated with migration dynamics and environmental influences, this analysis indicates levels of predictability, or connection strengths, between different variables in the system. We hypothesize that drivers of salmon migration dynamics will vary by season due to both environmental conditions and the number of salmon entering the system from the Pacific Ocean. Rather than analyzing any specific population of Chinook or a reservoir, we focus on nonlinear interactions between potential driving factors and salmon counts between distant dams throughout the Columbia River Basin. While we take a network approach to study one aspect of the salmon lifecycle, particularly drivers of migration timing, our framework is broadly applicable to explore dynamics in other environments subject to natural or human-induced variability.

## 2 Methods: Characterizing dependencies in the CRB system

We consider salmon dynamics and drivers based on two levels of analysis: *(a)* a 10-year study based on daily data from 2009-2018 to analyze short-term drivers of upstream migration timing and *(b)* a 35-year study based on annually resolved data from 1984-2018. For both analyses, we use daily video-based salmon counts. As the life-cycle of a Chinook is typically 4 to 5 years, the 10-year study captures several cohorts. Meanwhile, the dynamics based on daily information measures represent the effects of fluctuations in returning salmon counts, water temperature, and streamflows on migration timing rather than overall populations or survival. We note here that salmon may spend different lengths of time in the oceanic phase, such that returning salmon are from a combination of cohorts that may have experienced different environmental conditions over their lifespans. At a longer timescale considered in the 35-year analysis of annual data, these factors and others, such as ocean and freshwater conditions, can be cumulatively influential on annual salmon counts. For example, high salmon mortality in one season due to high temperatures can propagate to influence populations in subsequent years, or an altered flow regime could change future spawning behaviors, leading to variability in total annual counts. In this section, we first describe data sources and locations, followed by our information-theory based framework.

### 2.1 Salmon counts and environmental data sources

**Fig 2:**
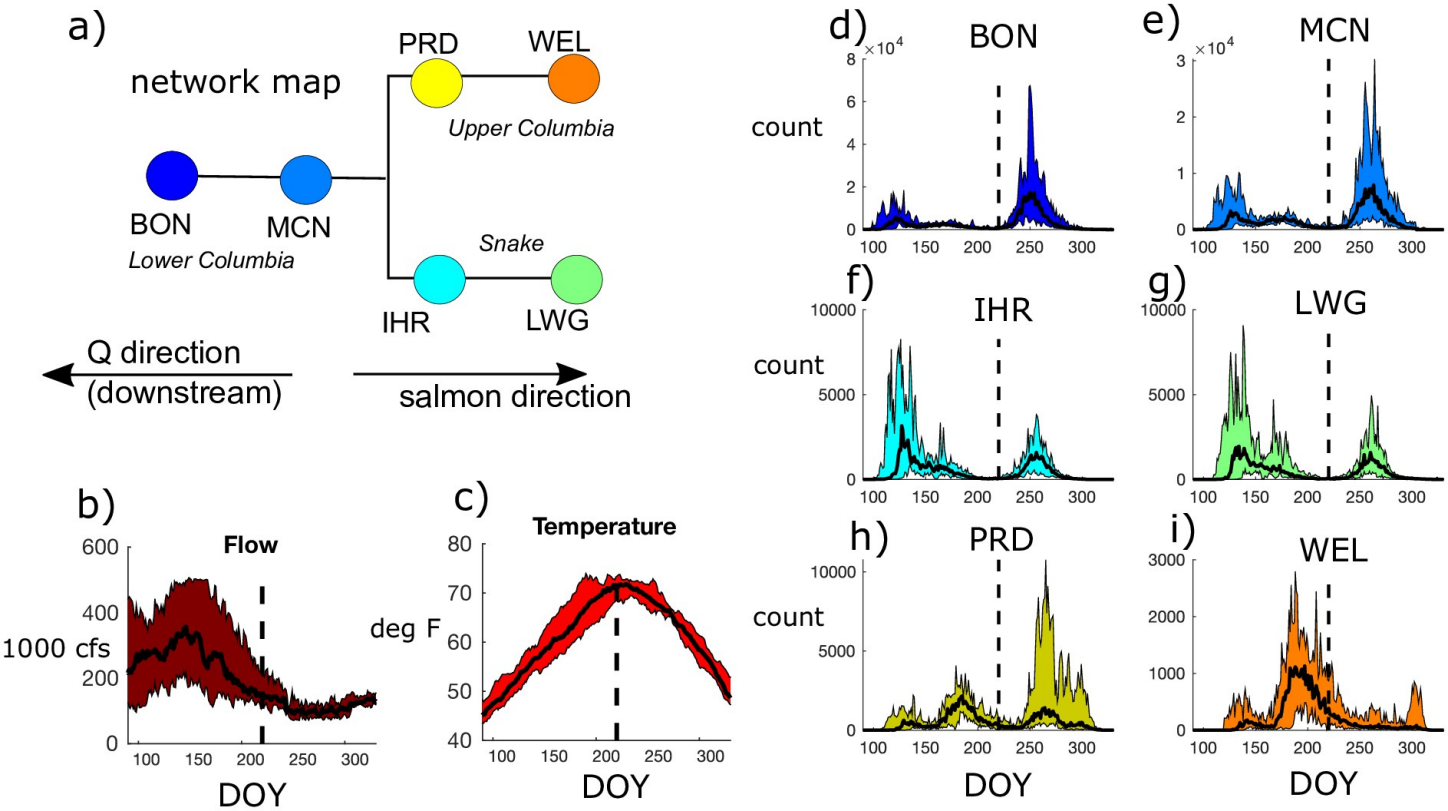
Distributions of salmon counts from day of year (DOY) 90 to 330 (early March through November) for 2009-2018. *(a)* An illustration of the network map of dams on the Lower Columbia, Upper Columbia, and Snake Rivers, where downstream flow rate is from right to left, while salmon migration is in the opposite direction. *(b-i)* Colored shading indicates minimum and maximum *(b)* flow rates and *(c)* temperatures at the Lower Columbia gage site, and *(d-i)* salmon counts for a given day. Solid black lines indicates the median values over the 10-year study period. The dotted vertical line in each plot separates defined spring (DOY 90-220, or March to August) and fall (DOY 221-330, or August to November) salmon runs. Note vertical axes have different ranges (e.g. maximum BON and MCN counts are higher than those at upstream dams).

We obtain adult Chinook counts from the Fish Passage Center [39] from 6 different dams on the Columbia and Snake Rivers: Bonneville (BON), McNary (MCN), Ice Harbor (IHR), Lower Granite (LWG), Priest Rapids (PRD), and Wells (WEL) (Fig 2a). These were chosen as they are the most upstream and downstream dams on the Lower Columbia, Snake, and Upper Columbia reaches. Each dam observes two different populations of adult Chinook returning to spawn, designated here as the “spring” and “fall” runs (Fig 2). The magnitudes of these runs differ between reaches due to regional geography and river conditions. In this study, we choose a specific day, DOY (day of year) 220, in early August, as the divide between the two runs. Although there could be overlap, and arrivals are lagged in time for the farthest upstream dams, we assume that the separate time-series datasets appropriately reflect the dominant dynamics in season. For example in this analysis, the spring run by default includes the “summer” run in the Snake (Fig 2f and 2g). The Snake River spring run is often referred to as the spring-summer run because it includes fish that return in early summer as well as in the spring. At the Lower Columbia dams, BON and MCN, the fall run is typically more significant. However, PRD, the first dam on the Upper Columbia, observes a larger median spring run. At WEL, the last passable dam on the Upper Columbia, the spring run is considerably lagged relative to BON due to travel times and the fall run is nearly non-existent in some years (Fig 2i). At this dam, it has previously been found that fallback and re-ascension at the fish passage causes more overestimates in salmon counts relative to lower elevation dams [40]. While we do not explicitly account for this source of error, it illustrates how uncertainties can vary between dams. The Snake River dams, IHR and LWG, have a more pronounced and highly variable spring/summer run. The major distinction between the dams with higher spring run counts (WEL, IHR, LWG, PRD) versus those with higher fall run counts (BON, MCN) is the location and elevation of the dams and the tributaries between segments.

We use video-based counts in this study instead of PIT-tag data that capture the trajectories of individual salmon, in order to apply information theory-based measures on a representative time-series. In other words, we consider overall lagged distributions of salmon counts and environmental indicators, rather than focusing on actual trajectories of a smaller number of salmon. This enables questions such as “how much does the knowledge of past flow rates reduce the uncertainty in the number of salmon passing a certain dam?” In contrast, tag data would provide insight into specific travel times and migration destinations, with fewer data points.

We use daily streamflows (*Q*) and water temperatures (*T*) at Lower Columbia, Upper Columbia, and Snake gage stations from USACE Northwestern Division [41] to compare with salmon counts. Since we find that the influences of flows and temperatures on salmon counts are similar between gage stations, we focus on the Lower Columbia gage to simplify the 10-year analysis of daily data (Fig 2b and 2c). While flow rates peak during the spring run (Fig 2b), water temperatures peak at the end of the spring run and the beginning of the fall run (Fig 2c).

For a longer term analysis of drivers and interdependencies related to salmon population abundances on multi-year timescales between 1984-2018, we also incorporate annual average water temperatures and flows, and annual cumulative precipitation and mean air temperature at the Hanford Site in Washington [42], located near the confluence of the Snake and Upper Columbia Rivers. We also consider the annual average Pacific Decadal Oscillation (PDO) index [43], and cumulative snow water equivalent (SWE) for four sites across the Columbia River Basin [44] as potential influences on salmon counts at the six dams (Table 1). Additional inputs derived from these data included the number of days a certain water or air temperature was exceeded (68 and 70°F for water and 90°F for air) at a site. 70°F is chosen as a relevant threshold for salmon health [5], while 68°F is used as a test to include water temperatures that approach the threshold. In our process network analysis, we consider all variables, including salmon counts, as potential lagged “sources” of information and salmon counts at upstream dams as potential “targets”. For example, lagged counts at BON are considered a potential source of information to counts at PRD, but not the other way around.

### 2.2 Information theory-based measures

We use information theory [47] measures to characterize information flows between lagged source and target variables. Here, target variables are salmon counts, and source variables could be salmon counts or environmental drivers (Table 1). Shannon Entropy measures the uncertainty of a random variable, *X*, and is defined as follows:

$$\begin{array}{c} H\left(X\right)=-\sum_{i}p\left(x_{i}\right)log_{2}p\left(x_{i}\right), \end{array}$$ (1)

where *p*(.) indicates a probability distribution over a variable, and the summation is over all possible states *x*_*i*_. The units of *H*(*X*) are *bits*, and can be interpreted as the average number of “yes” or “no” questions needed to determine the answer to a question, or the value of a random variable. We estimate probability distribution functions (*pdf*s) with a fixed bin approach with *N* = 11 equally spaced bins for the 10-year daily analysis, and *N* = 3 bins for the 35-year annual analysis due to more sparse data (35 data points per variable). The bins are spaced between minimum and maximum values for a given variable over the time period of record after extreme outliers have been removed. Since for the daily datasets, we consider fall and spring runs as separate time windows, this constitutes a “global” binning strategy in that the bins are defined over the same global range even though the range within a time window may be more narrow.

**Fig 3:**
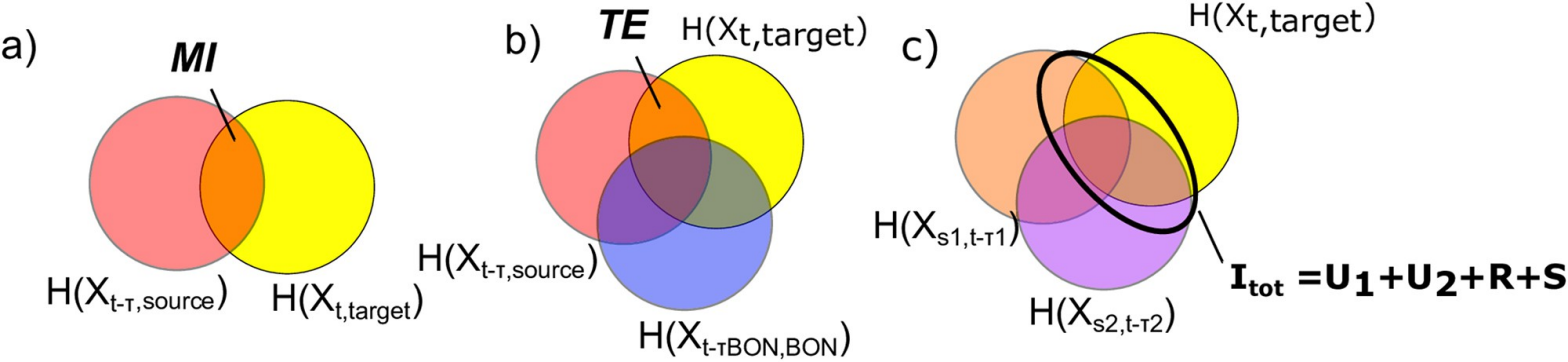
Venn diagrams illustrating range of information theory-based measures. *(a)* Mutual information (*MI*) between two random variables, where one is defined as a target and the other is a source that may be lagged in time. *(b)* Transfer Entropy (*TE*) between a source and target. Here we define *TE* as the *MI* conditioned on the knowledge of lagged salmon counts at BON. *(c)* Total mutual information, *I*_*tot*_, from two sources to a target is comprised of unique, synergistic, and redundant components.

Mutual information (*MI*) is the reduction in uncertainty of one random variable, given the knowledge of another (Fig 3a). Here we use *MI* to determine the extent to which the knowledge of a *τ*-lagged source (*X*_*s*,*t*−*τ*_), such as a downstream salmon count or environmental variable, reduces the uncertainty about a current target salmon count (*X*_*tar*,*t*_). We consider a lagged version of *MI* as follows:

$$\begin{array}{c} I\left(X_{s,t-\tau };X_{tar,t}\right)=H\left(X_{tar,t}\right)-H\left(X_{tar,t}|X_{s,t-\tau }\right)=\\ \sum p\left(x_{s,t-\tau },x_{tar,t}\right){\text{log}}_{2}(\frac{p(x_{s,t-\tau },x_{tar,t})}{p\left(x_{s,t-\tau }\right)p\left(x_{tar,t}\right)}), \end{array}$$ (2)

where the sum is a double summation over all source and target states. Here we define a dominant lag time, *τ* (units of days or years) as that which corresponds with the highest value of *MI* over a range of lags from 0-30 days in the daily analysis, and 0-7 years in the annual analysis. These lags ensure that enough data is available for robust estimations of lagged *pdf*s. As shown in Eq 2, mutual information is the difference between the entropy of a target variable, *H*(*X*_*tar*,*t*_), and the conditional entropy of that variable given the knowledge of another source, *H*(*X*_*tar*,*t*_|*X*_*s*,*t*−*τ*_) (Fig 3a). For the 10-year analysis of daily data we can next determine how source variables influence the target salmon counts jointly.

Transfer entropy (*TE*) [48] is a version of conditional mutual information, which measures the reduction in uncertainty of a target due to the knowledge of a source, beyond the uncertainty already reduced given the target history. Here we consider an altered version of *TE*, where we condition on the knowledge of the lagged salmon counts at BON instead of the immediate history of the target variable (Fig 3b). As any adult Chinook that reaches a dam in the CRB must first pass through BON, the counts at BON represent the total population that is “available” to the system. This conditioning also accounts for prior conditions in the marine environment that could impact salmon life cycles. For example, a weak observed mutual information relationship between water temperatures and salmon migration in a given season could be due to high ocean mortality that leads to fewer returning salmon. In this case, conditioning on the returning salmon with a transfer entropy measure makes the causal relationship between freshwater temperature and salmon counts more clear. We define *τBON* as the dominant lag time at which the salmon count at BON informs the salmon count at the upstream dam of interest (*X*_*tar*,*t*_) based on the *MI* measure in Eq 2. *TE* is then computed as follows:

$$\begin{array}{c} TE=I(X_{s,t-\tau };X_{tar,t}|X_{BON,t-\tau BON})\\ =\sum p(x_{s,t-\tau },x_{tar,t},x_{BON,t-\tau BON})\times \\ {\text{log}}_{2}(\frac{p(x_{s,t-\tau },x_{tar,t},x_{BON,t-\tau BON})}{p\left(x_{s,t-\tau },x_{tar,t}\right)p\left(x_{BON,t-\tau BON}\right)}) \end{array}$$ (3)

In addition to transfer entropy, we also consider the total information that different combinations of sources provide jointly to daily salmon counts at a given location. We particularly focus on counts at BON, flow rates (*Q*), and water temperatures (*T*), and compute total information as follows:

$$\begin{array}{c} I_{tot}=I\left(X_{s1,t-\tau 1},X_{s2,t-\tau 2};X_{tar,t}\right). \end{array}$$ (4)

Here, *X*_*s*1_ and *X*_*s*2_ are source variables defined as counts at BON, *Q*, or *T*. Lags *τ*1 and *τ*2 are the dominant time lags associated with the source variable based on *MI* individually. We note that *I*_*tot*_ is symmetric with respect to the two source variables, but not between a source and the target. In other words, the information that two sources provide to a target is not necessarily equal to the information that the target and one source provide to the other source. In an information decomposition, this total information quantity can be partitioned into four components as follows [49]:

$$\begin{array}{c} I_{tot}=U_{s1|s2}+U_{s2|s1}+R_{s1,s2}+S_{s1,s2} \end{array}$$ (5)

where *U*_*s*1|*s*2_ and *U*_*s*2|*s*1_ are information components that a source provides to the target *uniquely*, or individually, when the other source is also known. *R*_*s*1, *s*2_ is information that both sources provide *redundantly*, or in overlap, and *S*_*s*1, *s*2_ is information that sources provide *synergistically*, or jointly only when both sources are known together. Together, *I*_*tot*_ and the four information components provide a measure of total reduced uncertainty given the knowledge of two sources, in addition to features of the multivariate dependency (Fig 3c). For example, if one unique component is very high relative to other components, this would indicate that one source is a strong individual source of information to the target. Meanwhile, high redundancy indicates that the two sources are interdependent and provide overlapping information, and high synergy would indicate that the two sources predict the target only when known jointly. Other information theory measures also can be expressed in terms of information decomposition components [49]. Specifically, both *MI* and *TE*, which are used in this study, capture a *unique* information component between the source and the target, which here is information that is not also provided by knowing the lagged count at BON. In addition to this unique aspect, *MI* contains a *redundant* component, or overlap in information that both the source and the lagged returning salmon from BON provide to the target site. In contrast, *TE* captures a *synergistic* component, or information only obtained when both sources are known together. For example, if *MI* > *TE* for a given source-target pair, the conditioning in *TE* “explains away” some of the observed dependency based on *MI*, and we know *R* > *S*. If *MI* < *TE*, the conditioning actually enhances the observed dependency by incorporating extra, or synergistic, information (*S* > *R*).

These unique, synergistic, and redundant components are useful constructs with which to interpret joint dependencies, but information theory does not provide a direct method to compute them and multiple methods have been proposed. We use a re-scaled redundancy metric [27] to compute redundancy, or the “overlap” in information between two sources. This re-scaled metric considers that redundancy is upper and lower bounded by information theory, and can be scaled between these bounds based on the mutual information (*MI*) between the two source variables. Specifically, two source variables that have a high *MI* lead to maximally high redundancy, while independent source variables (*MI* = 0) lead to minimal redundancy. We refer to [27] for detailed methodology and example applications of this measure. A redundancy measure such as this, in addition to known information theoretic relationships, enables us to compute the other measures of uniqueness (one from each source) and synergy. Hereafter, when the sources, *s1* and *s2*, are defined, we remove the subscripts and refer to information components as *S*, *R*, *U*_*s*1_, and *U*_*s*2_ to simplify notation.

For the 35-year analysis of annual total salmon counts, we only compute lagged *MI*, and do not consider higher order measures such as *TE* or information decomposition. Instead, to study multi-year dependencies between environmental drivers and adult salmon abundances rather than migration dynamics within seasons, we normalize annual total salmon counts upstream of BON by counts at BON, such that MCN, LWG, IHR, PRD, and WEL in the 35-year study represent fractions of salmon from BON that arrive at each dam in a given year. This enables us to account for the number of salmon entering the system without computing higher order measures, as we have fewer data points for information theory computations that involve 3D *pdf*s. In other words, higher order information theory measures have larger data requirements in order to obtain robust results, so we restrict this analysis to two-dimensional measures. While other studies aim to determine the best combination of variables to predict salmon returns for a given year [17], we focus on non-linear relationships between pairs of variables in our longer term analysis of annual totals.

We normalize *TE*, *MI*, and *I*_*tot*_ measures by the entropy of the target variable, *H*(*X*_*tar*,*t*_), such that each information measure indicates the fraction of total uncertainty that is reduced. For statistical significance testing of any given information measure, we perform 100 shuffled surrogate tests in which the target time-series data are randomly shuffled to remove time correlations between the sources and target [26, 28]. This shuffling results in no change in entropy of an individual variable, but typically a decrease in shared information, or de-coupling, between the multiple variables. We compute the relevant information theory measure (*MI*, *TE*, or *I*_*tot*_) using the shuffled datasets, and a critical information value is defined as *I*_*crit*_ = *I*_*shuff,mean*_ + 3*I*_*shuff,stdev*_. Any information measure that is below this critical threshold is determined to be non-statistically significant and is set to zero. In other words, we test observed information measures against measures computed based on many iterations of randomized data with the same individual distribution. While there are confounding factors and inherent uncertainties in salmon count data such that no combination of predictive factors can fully reduce uncertainty, we assume that statistically significant information measures indicate a predictive relationship that is potentially causal. For both the 10-year study using daily data and the 35-year study using annual values, we compare values of entropy and mutual information with linear counterparts of the coefficient of variation ($CV=\frac{\sigma }{\mu }$) and the correlation coefficient, respectively. While it is expected that a high correlation between two variables generally corresponds to high *MI*, we note that this is not always the case since *MI* may be less sensitive to linear correlations due to binning for *pdf* estimations, but also captures non-linear relationships that would not be detected as correlations. Meanwhile, there are no linear comparisons for IT-based measures of *TE* or information decomposition, as these are based on multivariate distributions of source and target variables.

## 3 Results: Seasonally varying Chinook drivers

For daily salmon counts of fall and spring runs from 2009-2018, we find that entropies are higher for fall run salmon counts in the Lower Columbia River, spring run counts in the Snake River, and vary for the Upper Columbia sites (Table 2). These higher entropies correspond with the season with larger ranges of counts for each dam location (Fig 2). For example, the lower Columbia dams, BON and MCN, have wide ranges of counts in the fall relative to the spring (Fig 2d and 2e), corresponding to high entropy. The coefficient of variation (*CV*) for salmon counts shows a similar pattern, except for IHR and PRD, the most downstream sites on the Snake and Upper Columbia Rivers, respectively. This illustrates that these measures of variability are not directly comparable and depend on the shape of the distribution. Meanwhile, both entropy and *CV* for flow rate and water temperature are considerably higher for the spring run relative to the fall. Since entropy represents uncertainty, these results indicate that most variables are more difficult to predict on a day to day basis during the spring run.

**Table 2:** Entropy (*H*(*X*)) in units of *bits* for fall and spring runs for daily salmon counts at six dams (listed by site name) and flow rate (*Q*) and water temperature (*T*) in the Lower Columbia River, along with the coefficient of variation (*CV*) for comparison. The largest value of the two time windows is highlighted in bold for each category.

	spring run *H*(*X*)	fall run *H*(*X*)	spring run *CV*	fall run *CV*
BON (daily counts)	2.31	**3.16**	0.21	**0.35**
MCN (daily counts)	2.68	**2.93**	0.35	**0.55**
IHR (daily counts)	**3.21**	3.04	0.43	**0.60**
LWG (daily counts)	**3.16**	3.13	**0.53**	0.41
PRD (daily counts)	2.79	**2.92**	**0.51**	0.42
WEL (daily counts)	**2.95**	2.89	**0.70**	0.47
Q (cfs, daily)	**3.18**	1.37	**0.37**	0.22
T (deg C, daily)	**3.05**	2.77	**0.14**	0.11

### 3.1 Dependencies between salmon counts

**Fig 4:**
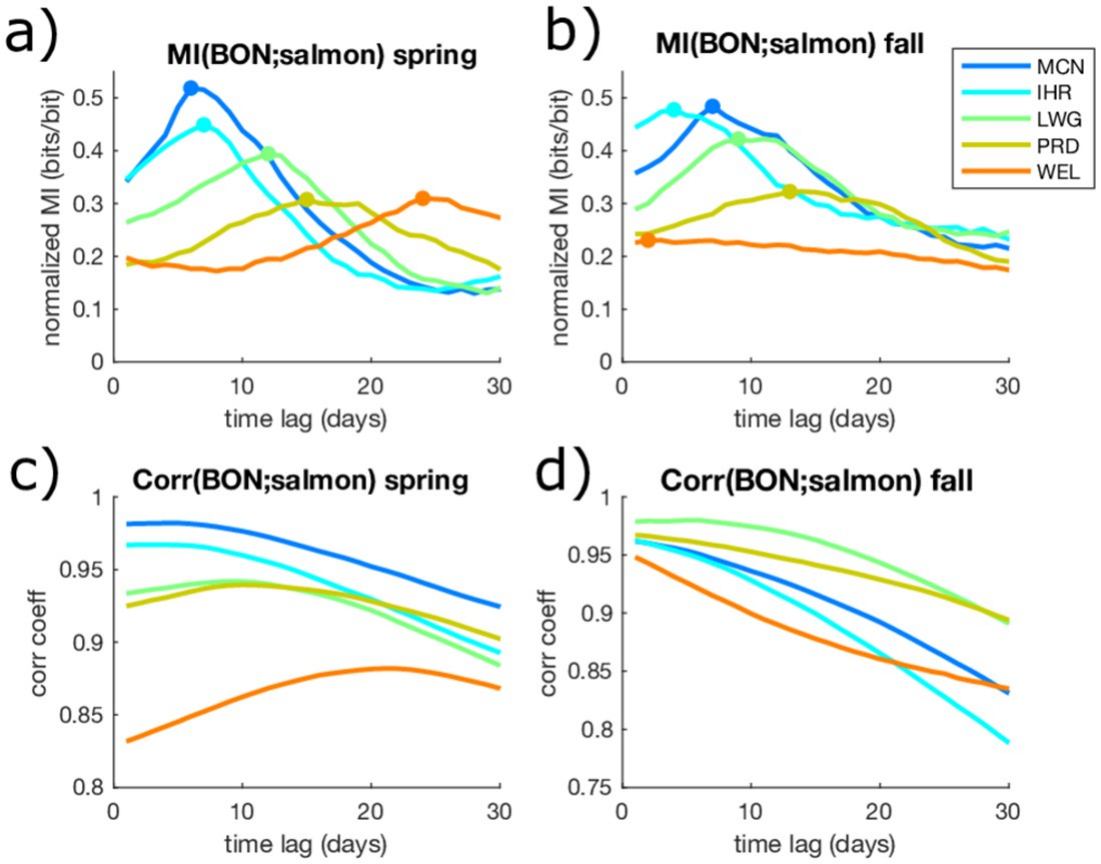
Mutual information *(MI) (a,b)* and linear correlation *(Corr) (c,d)* between BON daily salmon counts and counts at upstream dams for spring run *(a,c)* and fall run *(b,d)* salmon, for time lags, *τ*, from 0 to 30 days. Dots indicate lag times associated with peak *MI*, which are used as *τ*_*BON*_ in *TE* computations for conditioning on knowledge of lagged salmon counts at BON.

Salmon counts at a given dam tend to be most tightly connected with those at their nearest downstream neighbor and BON, where salmon enter the CRB network. Peak *MI* decreases with greater distance between BON and a given dam, indicating that distance traveled leads to a loss of information in Chinook counts between dams (Fig 4). The dominant lag, *τ*, associated with peak *MI*, can also be associated with travel time between dams, and we see that the dominant *τ* tends to increase with travel distance from BON (Fig 4). MCN and IHR, the two closest dams to BON, both have sharp peaks with a well-defined dominant *τ*, particularly for spring run salmon. WEL, the farthest dam from BON, has a much more gradual peak, which can be attributed to compounding impacts from fluctuations in river dynamics along the reach. In other words, there is greater probability of exposure to factors that effect salmon with distance. These cause salmon to be more distributed over a longer distance and arrival timings are more variable. At WEL, it has been found that migration delays do not necessarily lead to higher mortality [40], but timing may impact other life stages, such as spawning behaviors. For instance, cumulative thermal exposure has been found to be strongly connected to migration duration, and salmon may pause at thermal refuges near tributary entrances in extreme temperature conditions [50]. For Chinook traveling great distances such as those that arrive at WEL, these environmental influences have more time to act upon the migrating Chinook, resulting in more spread in the timescales that we observe here. We also compare *MI* with lagged correlations between salmon counts and upstream dams (Fig 4c and 4d). The lagged correlations show some similar patterns as *MI*, but the peaks are less distinct and correlation values are grouped more closely together. In general, lagged correlations are similarly statistically significant, but *MI* distinguishes peak dominant lag times more clearly.

**Fig 5:**
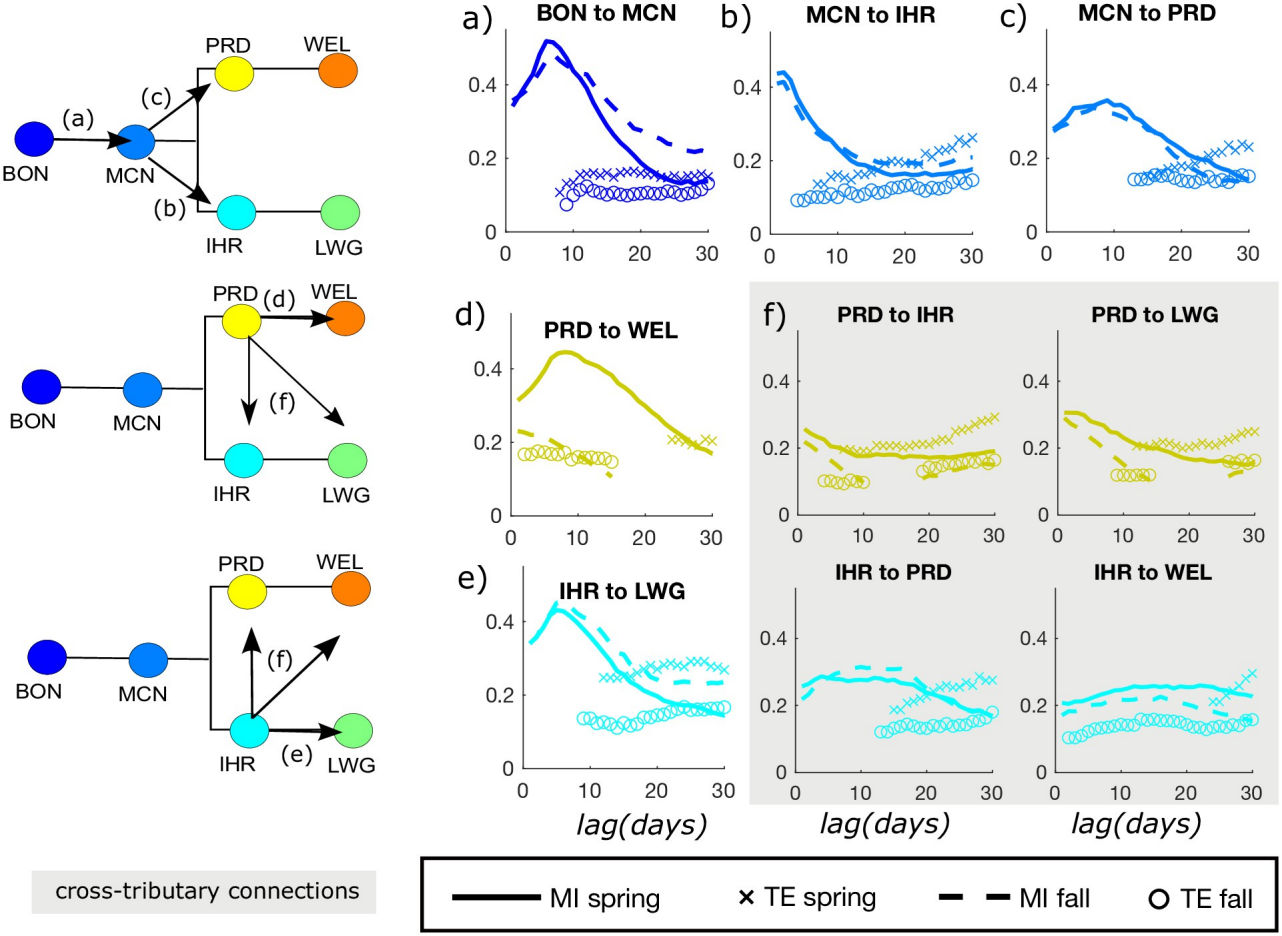
Downstream to upstream *(a-e)* and cross-tributary *(f)* *MI* and *TE* (units of *bits/bit*) between daily salmon counts. Network map icons on the left side of the figure indicate links associated with each plot on the right. In each plot, the horizontal axis indicates the time lag, *τ* (days) between the source (the downstream dam) and target (upstream dam), and the vertical axis is the fraction of reduced uncertainty of salmon counts at a given dam, or *MI* (or *TE*) divided by the total uncertainty, *H*(*X*_*tar*,*t*_), shown in Table 2. Since *TE* is conditioned based on dominant time lags between the target dam and BON (shown in Fig 4), values of *TE* are not computed until that lag time. Otherwise, gaps indicate non-statistically significant values. Gray shaded plots *(f)* indicate cross-tributary connections, where salmon do not actually migrate from one dam to another.

Lagged salmon counts directly downstream of a target dam often provide high *MI*, but the magnitude is run-specific (Fig 5a–5e). On the Upper Columbia, *MI* between counts at PRD and WEL for fall is very low, and higher for the spring run (Fig 5d). In contrast, at the Snake River dams, IHR and LWG, *MI* between counts is similar for fall and spring runs, but *TE* is higher for spring run salmon. (Fig 5e). In general, cross-tributary *MI* connections (Fig 5f) are weaker than connections between neighboring dams. For these cross-tributary connections where salmon are not physically migrating from the source to the target site, we see that *MI* has less of a distinct peak, but is often statistically significant along with *TE* at multiple lag times. Particularly in the spring, *TE* is greater than *MI* at longer time lags, indicating that there are some predictive relationships between cross-tributary salmon counts that are enhanced given the knowledge of lagged counts at BON. While this predictive relationship cannot be construed as causal, it does indicate connectivity between the Upper Columbia and Snake River salmon counts. This could relate to the mass balance relationship between these two major tributaries and the relative timing of Chinook passage at the dams. Specifically, when Chinook pass BON, the portion that reaches the confluence of the Snake and Upper Columbia Rivers is split between spawning in lower Columbia tributaries or migrating toward either PRD or IHR. This could create an inverse type of relationship between these counts when also considering the lagged count at BON. In other words, if a Chinook that passed BON reaches IHR, it becomes (relatively more) certain that it will not reach PRD. As the travel time, estimated by the dominant lag of *MI*, from BON to IHR is considerably shorter than from BON to PRD in both fall and spring runs (Fig 4), we find that knowing the counts on the Snake at IHR reduces the uncertainty of future counts on the Upper Columbia at PRD in the coming days.

*MI* also differs between seasons for the dams at the confluence of the Snake (IHR) and Upper Columbia (PRD) with respect to the Lower Columbia (MCN). MCN is similarly predictive of IHR and PRD counts in spring and fall runs (Fig 5b and 5c). However, this is not true for the Lower and Upper Columbia dams farther from the confluence. For example, lagged *MI* from PRD to WEL is low in the fall run (Fig 5d). In this way, the low *MI* between counts at BON and WEL during the fall can actually be attributed to low *MI* between counts at PRD and WEL along the Upper Columbia, and is not particularly related to dynamics at the confluence. We also note that spring run Chinook prefer to spawn in the high elevation tributaries (many of which are located past WEL) due to the high flow and low temperature during this time [40], while the fall run favors the lower elevation streams prior to WEL.

### 3.2 Flows and temperatures as drivers

**Fig 6:**
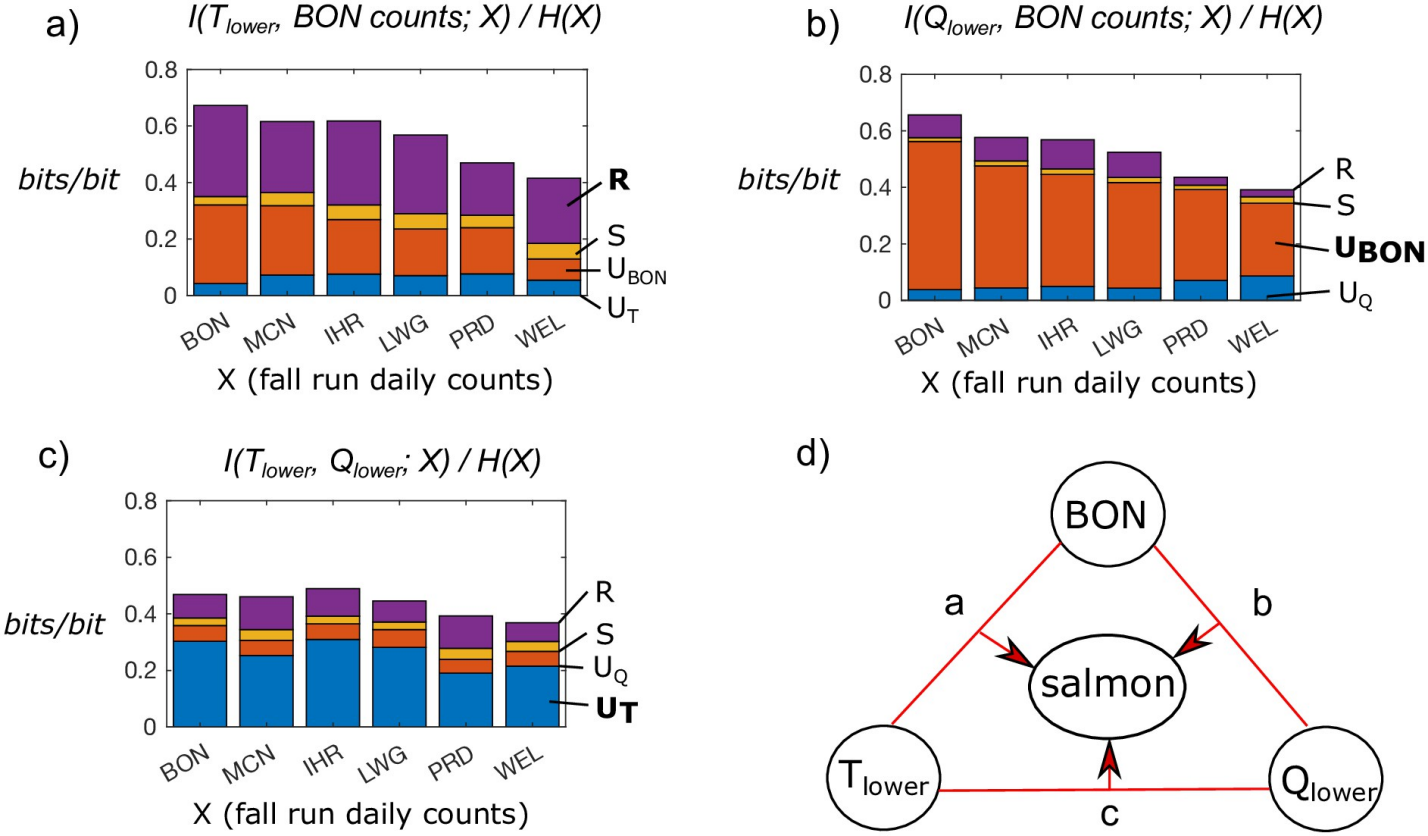
Total information (Eq 4), normalized by entropy of salmon counts, and information decomposition components (Eq 5) to counts at each dam, from combinations of two joint sources for fall run salmon counts. Bar heights indicate total information, while colors are proportions of unique, synergistic, or redundant information types. *(a)* Lower Columbia water temperature *T*_*lower*_ and counts at BON as lagged information sources. *(b)* Lower Columbia flow rate *Q*_*lower*_ and BON as lagged information sources. *(c)* *T*_*lower*_ and *Q*_*lower*_ as lagged information sources. Patterns of joint information are similar for spring run salmon, but some information from *Q* was found to be non-statistically significant.

When we view lagged *T*, *Q*, and counts at BON as pairs of potentially joint predictors of salmon counts (Fig 6), we see that pairs of sources provide unique, redundant, and synergistic information to counts3.2 at a given location to different extents. For all pairs of variables, we see that predictability, in the form of $\frac{I_{tot}}{H}$, tends to decrease for upstream dams, especially PRD and WEL on the Upper Columbia. The predictability of salmon at WEL in the fall is particularly low (about 0.4 *bits/bit*, or 40% of total uncertainty) given any pair of source variables. Meanwhile, the predictability of counts given lagged BON and *T* is close to 60% for the downstream dams (Fig 6a). In Fig 6 we only show information partitioning results for the fall run, since spring run partitioning results are extremely similar except that information flows from *Q* to salmon counts at some dams are not statistically significant. While flow rates are more highly variable in the spring as shown from higher entropies (Table 2), this variability causes the predictive relationship between flows and salmon counts to be weaker.

*T* and BON provide the most redundant information relative to other pairs of variables (*T* and *Q*, BON and *Q*). While the amounts of redundant and unique information from *T* remain similar between upstream and downstream dams, unique information from counts at BON decreases with distance from BON. We note that unique information from *T* is lowest at BON (Fig 6a), which is expected since salmon passing BON are unlikely to have been impacted by freshwater temperatures. We see a similar pattern of decreasing predictability for upstream dams for the combination of sources of *Q* and BON, but here the main information component is unique information from BON, indicating that *Q* is a much weaker source (Fig 6b). However, the unique information from *Q* to counts increases from downstream to upstream, and *U*_*Q*_ is highest for WEL. Meanwhile, synergistic and redundant information are relatively small. Finally for the combination of *T* and *Q*, *T* is typically the strongest unique source, and redundancy and synergy are both relatively low (Fig 6c). For the combination of *Q* and *T* as information sources to counts, we see less of a pattern of decreasing information with distance from BON, and the joint predictability peaks for counts at IHR, the most downstream dam on the Snake River.

While here we only compare pairs of sources rather than many joint sources in order to maintain a low dimensionality of information theory measures, we can compare the three sets of pairs and make several inferences about their three-way interaction as predictors of counts. For instance, we see that *T* tends to be the strongest predictor of counts relative to both BON counts and *Q*. However, the level of predictability from *Q* and BON together is similar or greater than unique, or individual, information from *T*. This highlights that water temperature is a strong individual predictor of salmon counts in the Upper Columbia, but lagged *Q* and BON are similarly strong predictors when they are accounted for jointly. The time lags associated with lagged BON counts for each dam are highlighted in Fig 4, and we do not specifically discuss dominant time lags associated with mutual information from *Q* and *T* at the different dams as they are less correlated to geographical distance and mutual information varies less over the range of lag times. In other words, lagged counts at BON are most predictive of salmon counts when considered at a very particular time lag, but *Q* and *T* provide similar information quantities at a range of time lags. This indicates the presence of long term memory in flow and temperature variables that does not exist in salmon counts.

### 3.3 Process networks based on daily data

**Fig 7:**
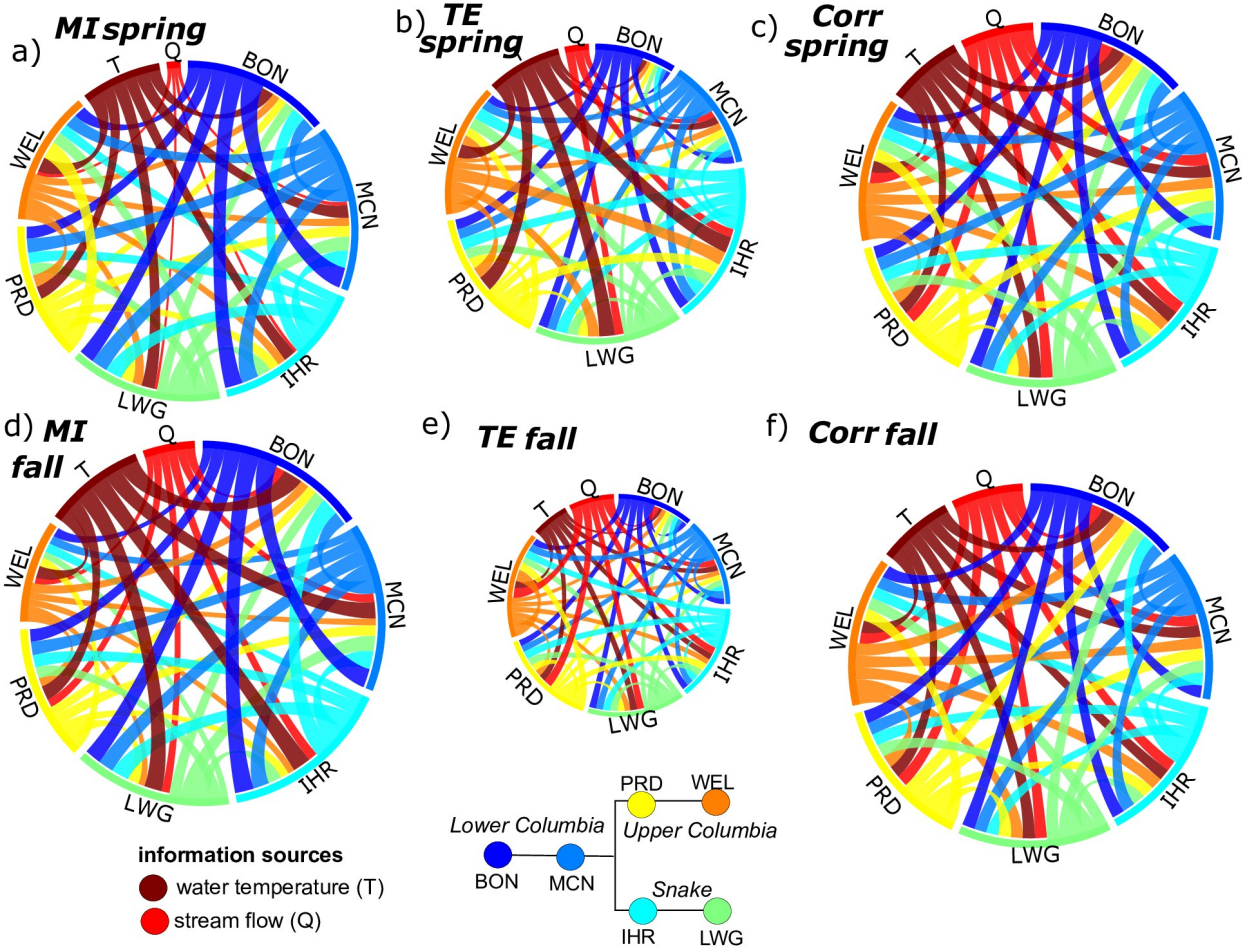
Mutual information *MI (a,d)*, Transfer Entropy *TE (b,e)*, and linear correlation *Corr (c,f)* links between salmon counts at dams, flow rate (*Q*), and water temperature (*T*) for spring run *(a-c)* and fall run salmon *(d-f)*. *Q* and *T* correspond to Lower Columbia data only, as strengths are similar for other *Q* and *T* variables. Link widths for *MI* and *TE* indicate relative information strengths, and colors correspond to information sources.

Here we present an analysis of a process network, which involves the entire range of pairwise connections between salmon counts, water temperature, and flow rate based on daily data. Here we find that normalized *MI* values range from 0.07—0.47 in the spring run and from 0.07-0.57 in the fall run. Since normalized *MI* ranges from 0 to 1, this indicates that lagged predictor variables span a relatively wide range of strengths in terms of the information they provide. Particularly, the knowledge of lagged salmon counts or flow and temperature conditions can reduce uncertainty of salmon counts at a given dam by up to 57%, and on average we find that any given source variable reduces about 20% of uncertainty. Fig 7 shows the statistically significant linkages between salmon counts, temperature, and flows in terms of *MI* and *TE*, for spring and fall runs, in addition to lagged linear correlations. As found in previous studies focusing on marine survival [19], the complete life-cycle [3], or adult returns [17], this indicates a high level of connectivity within the system, and no single variable is likely to explain more than half of the observational uncertainty. While *TE* < *MI* overall (circle sizes and link widths in Fig 7), the higher *TE* in spring indicates less redundancy, and more unique drivers of salmon counts during that season. Meanwhile, lagged linear correlations (Fig 7c and 7f) are all relatively similar in magnitudes, and similar across seasons, such that they do not highlight any particularly strong drivers or differences between salmon runs.

*MI* between Chinook counts at BON and the upstream dams is higher in the fall run relative to spring for every dam except WEL (Fig 7a and 7b, peaks in Fig 4). Lower variability in streamflow in the fall run (Fig 2b, Table 2) likely contributes to the increase in peak *MI* between counts at BON and most dams during this run, since streamflow is a less important factor in upstream migration. Meanwhile, the smaller magnitude of the fall run for Chinook at WEL (Fig 2i) causes the lower *MI* for that case. Water temperatures (*T*) appear to be stronger drivers of Chinook counts relative to flows (*Q*) based on *MI* in both spring and fall runs (Fig 7a and 7b). This is in agreement with a previous finding that salmon migrate fastest at an optimal temperature, and flow velocity is a less significant driver [51]. Higher water temperatures can particularly cause salmon to delay their migration and use thermal refuges [50, 52], which may cause a strong relationship between lagged temperatures and salmon counts. Meanwhile, low flows are generally linked with higher temperatures, and high flows are associated with bed scour which can influence salmon, but on a more lagged time-scale since these high flow events occur in early spring [53]. While the influence of bed scour is likely to be very weak for adult salmon, this illustrates how high flow rates may be either beneficial or detrimental to migrating salmon.

Several key aspects of seasonal and geographic variability are summarized as follows, and illustrated in Fig 7:

1. *MI* between salmon counts at BON and upstream dams decreases with distance from BON, while associated lag times increase (Fig 4). This *MI* is higher in the fall run relative to spring for every dam except WEL.
2. *MI* between counts at neighboring dams is generally higher than *MI* between counts at more distant dams, but some cross-tributary connection strengths between salmon counts are statistically significant, indicating predictive relationships. We also detect relatively weak but statistically significant connections from upstream to downstream salmon counts, which are opposite to the direction of migration and cannot be construed as causal.
3. *TE* < *MI* for dependencies between salmon counts, indicating that the knowledge of lagged counts at BON provides redundant information that tends to partially explain observed dependencies between counts at other sites.
4. *MI* from water temperature (*T*) to salmon counts is higher than *MI* from streamflow (*Q*) to salmon counts, particularly for the spring run.

## 4 Results: 35-year study (1984-2018)

**Fig 8:**
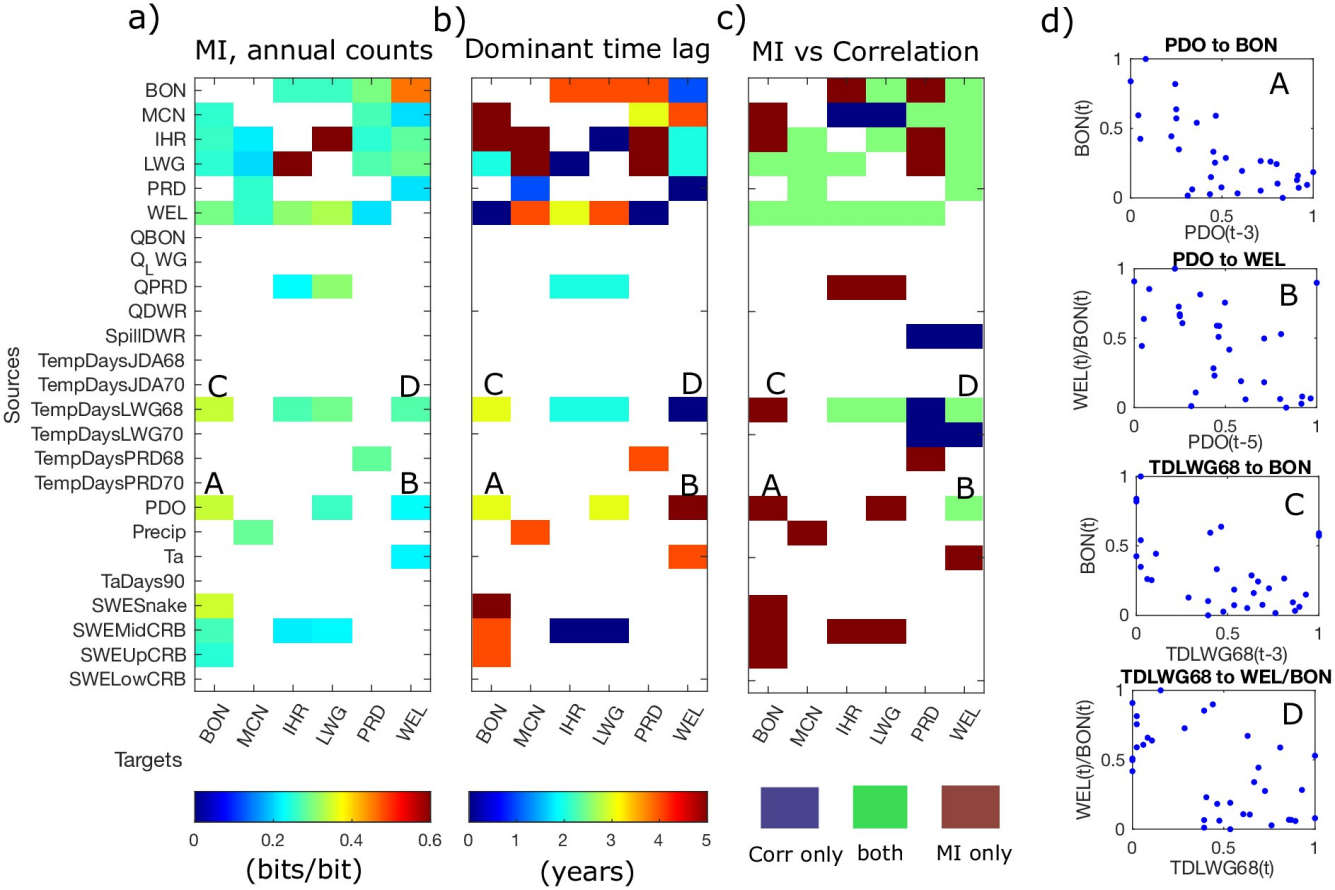
*MI* and dominant lags between indicator variables and annual total counts for 35-year windows, and comparison of statistically significant links based on *MI* versus linear correlation. *(a)* Mutual information divided by entropy of annual salmon counts. *(b)* Associated dominant time lags (years) for each *MI* measure. *(c)* Statistically significant (*p* < 0.01) detections of *MI*, linear correlation, or both for each variable pair. White areas indicate pairs for which neither *MI* or correlations are detected. *(d)* Scatter plots of certain annual variables, normalized to a 0-1 range, showing lagged relationships for selected source-target pairs labeled in *(a-c)*, illustrating both linear and non-linear dependencies captured as *MI* (B,D) or both linear correlations and *MI* (A,C).

Of the 25 annual variables that we consider as potential sources of information in the longer term study, nine (and the counts themselves) are found to be predictive of annual salmon counts in the form of statistically significant *MI* (Fig 8a, and bold variables in Table 1), where statistical significance testing is described in the methods section. These are the PDO, precipitation (Precip), air temperature (Ta), snow water equivalent (SWE) measured at most locations within the basin, flow rate at PRD (*Q*_*PRD*_), spill from the Dworshack dam (Sp_DWR_), and degree days above 68 at LWG (TD_LWG68_).

The most tightly synchronized salmon counts, on an annual basis, are IHR and LWG, located on the Snake River (Fig 8a). Annual totals at these dams share mutual information that peaks at an instantaneous (zero-lag) time scale (Fig 8b). This dependency between the two neighboring sites is further confirmed by a correlation analysis (Fig 8c), indicating that the relationship is somewhat linear. Counts at dams also share statistically significant *MI* with counts at most other dams, in addition to being linearly correlated. For example, the knowledge of salmon counts at BON in one year are highly predictive of normalized counts at WEL the following year. In general however, lag times associated with peak *MI* vary from 0 to 5 years. We note that *MI*-based connectivity between neighboring dams or between tributaries does not indicate casual relationships, particularly since no conditioning is involved, but it does indicate predictive relationships that arise due to spatial synchrony of weather and climate conditions across the basin in addition to salmon life cycles. While the time lag analysis based on daily data clearly reflects the migration timing of salmon, dominant lag times between counts at different dams show less of a clear pattern at the annual scale.

Other dependencies between environmental variables and salmon counts are more likely to be captured by either statistically significant *MI* or correlations, but not necessarily both (Fig 8c). This indicates that given a small sample size (35 years of annual data), information theory-based measures may not capture linear relationships as strongly as correlations, but they still detect other dependencies. Of all environmental drivers, TD_LWG68_, the number of days with water temperatures above 68 degrees F at LWG on the Snake River, has the most correlations and *MI* with salmon counts. Panels C and D of Fig 8d show the actual data points for one case where only *MI* is detected and another where both *MI* and correlation is detected. From a visual inspection however, it appears that for both cases, salmon counts are negatively correlated with TD_LWG68_, indicating that higher water temperatures, particularly along the Snake River, are associated with lower salmon counts either within a year or over a several year window. TD_LWG68_ is also the only environmental source variable that provides information to salmon counts in all three major tributaries (Fig 8a). This shows that water temperature is a major factor in Chinook dynamics for both instantaneous interactions, as it influences migrating adult Chinook within a season and over multiple years. Within a year, water temperature can lead to higher disease, parasites, higher predation rates, and poorer ability to evade predators, and across years it may influence salmon over different life stages due to carryover effects [18].

The mutual information relationship between lagged PDO and BON salmon counts is relatively strong (Fig 8a, A), but weaker between the PDO and upstream salmon counts normalized by BON counts. In other words, dividing annual salmon counts by counts at BON partially omits the ocean influence, indicating that ocean conditions influence the number of salmon that reach BON, but does not further influence salmon once they reach that location. The PDO also has a negative correlation with BON Chinook counts (Panel A of Fig 8), in agreement with the relationship initially discovered [54] and other recent findings [55]. The dominant lag of several years approximately corresponds to the oceanic growth phase of a Chinook life cycle, which is when a population would most likely be impacted by oceanic conditions.

Finally, *MI* from precipitation (Precip) and snow water equivalent (SWE) at several sites across the basin are also statistically significant at lags of 3 years or more, indicating weather-related impacts to juvenile Chinook that then propagate to returning adult populations. Precip is only predictive of fractional counts at MCN, and only based on *MI*. This likely reflects management in the basin, that results in an indirect relationship between precipitation and flows that varies depending on seasonal timing and whether the precipitation is rain or snow. For example, precipitation as rainfall allows ground pollutants to enter the river system, which can result in mass mortality events for salmon [56]. In this way, precipitation can have a positive or negative effect on salmon populations, depending on timing relative to migratory seasons and rain versus snow conditions. This type of relationship precludes any detection of a linear correlation, but an information measure may still detect it.

## 5 Discussion and conclusions

Our findings align with several known interactions in this system, and also highlight novel aspects due to our process network framework. For example, the PDO and water temperature are known to significantly impact Chinook population dynamics [21]. Accordingly, we find that the knowledge of water temperature-related variables reduces the uncertainty of salmon counts at both seasonal and multi-year timescales, and the PDO reduces count uncertainty at lags similar to the oceanic phase of a salmon life span. We also find that the connections between salmon counts across tributaries of the Snake and Upper Columbia reaches, in addition to environmental drivers that span across the basin, indicate correlations across the large geographical region of the Pacific Northwest, as found in other studies [17]. A recent synthesis of declining salmon populations [57] determined that Pacific salmon smolt-to-adult return ratios (SARs) have declined to one third of their historic size, but this decline is similar across basins, indicating that other factors are at play besides reservoir management. Additionally, it has been found that managed pulse flows are not always significant drivers of salmon migration [13], indicating the presence of multiple and nonlinear drivers that can vary between years [20]. However, migration timing is an indicator of cumulative salmon stress levels, and is influenced by both human management of reservoir systems and climate change [3]. Results presented here confirm that salmon migrations are influenced by multiple freshwater habitat-based drivers on both long and short timescales, in addition to ocean conditions. We also show that the knowledge of sources jointly can provide similar information about salmon counts as a single stronger individual source. In models, these input combinations, whether they are predictive due to correlative or causal relationships, are important to consider.

An information theory-based approach such this enables the detection of joint interactions in addition to nonlinear dependencies relative to other techniques such as regression models based on linear correlations. In terms of joint interactions, we find that lagged streamflows provide information to salmon counts, but the predictive relationship is stronger when we condition on salmon entering the basin. For example, the knowledge of streamflow and counts at BON at certain lag times provide synergistic information about upstream counts, which indicates that there is some predictability that could not have been realized given the knowledge of those sources individually. While we focus on levels and thresholds of predictability rather than the development of a specific predictive model, this provides a useful framework with which to compare model hypotheses, based on their representations of observed interactions [58]. In this way, the framework presented here is useful for model development, in terms of selecting predictors that may have non-linear or joint relationships with the target output, or for developing hypotheses that can be tested by new modeling approaches. The ability of information theory-based measures to characterize nonlinear dependencies also differentiates our framework from techniques based on variances or linear correlations. For example, while a mutual information (*MI*) measure does not specify whether a value indicates a “positive” or “negative” correlation, mutual information captures relationships that would not be detected at all based on the correlation measure, as shown in the 35-year example based on annual variables. While “process connectivity” using similar information measures has been explored in hydrology and ecohydrology contexts [29, 32], this is the first application to an aspect of the life cycle of a species. In general, this framework could be extended to explore nonlinear and multivariate interactions for different parts of salmon life cycles to further study carry-over effects, model predictive skill, and shifting drivers of ocean and freshwater survival.

Additional variables of interest that we did not consider include total dissolved gasses (TDG) and spill from multiple dams, juvenile Chinook counts, other salmon species, or weather at a higher spatial resolution. These variables also impact water quantity and quality, and could impact migration. A more detailed analysis that incorporates additional spatial and temporal variability could detect drivers at a specific location, such as a particular dam location that is subject to spills, has certain areas for refuge, or has inflows that influence water temperature and quality. There are also timescales that are important to salmon, such as monthly lags that we did not account for in this study that focused on adult migrating salmon at daily and annual scales. For examples, winter snowfall has an influence on water temperatures the following spring, and ocean temperatures could have an influence on salmon entering the CRB on a weekly to monthly timescale. Another source of uncertainty in our annual analysis is the combination of fall and spring runs into the annual total salmon counts. Here, the fall and spring salmon populations could have different drivers that we do not distinguish. There are also sources of error and limitations in the salmon count dataset, and other methods exist to track salmon migrations [59]. For example, there is considerable debate regarding the best way to accurately track drivers of salmon mortality based on different measurement or tagging techniques [22, 60]. The video-based counts used in this study represent upstream escapement rather than abundances, and do not distinguish between hatchery and wild salmon. The counts do not account for salmon that are transported or harvested between dams along the route, and also include uncertainties due to overshoot and fallback, which cause overestimates in the counts. For example, salmon that were transported as juveniles may exhibit different behavior in terms of their likelihood to home to their natal stream. These features of fisheries, trap-and-haul operations and fallback are therefore embedded into the salmon count data and a more detailed analysis would be needed to separate these influences. However, we assume that daily video-based counts generally reflect the upstream migration timing of adult Chinook, rather than the trajectories of individual salmon which can be obtained with PIT tagged data. While this migration timing does not directly link to survival because salmon are somewhat adaptable to changes in climate or obstacles such as dam passage fallback and re-ascension [61], it does relate to cumulative stress and impacts other life stages. More specific datasets such as tagged salmon counts, smolt-to-adult return ratios (SARs) [57], and fish passage success [61] that are available for certain dams and time windows could be integrated to further disentangle complex drivers throughout the salmon life cycle.

A recent report released by multiple agencies outlined several goals and operational measures to mitigate the decline in salmon populations in the Columbia River Basin [62]. Understanding magnitudes and geographic characteristics of interactions between Chinook populations and environmental drivers is essential to decision-making in these ongoing recovery efforts. For example, the PDO likely switched into a warm phase around 2015, which is accompanied by decreases in Chinook populations across the Pacific Northwest [46]. Moreover, climate change in the Pacific Northwest is expected to involve regional air temperatures increases of 0.1 to 0.6° C per decade which will increase the number of extreme water temperature events [63, 64]. Under human management decisions and future climate uncertainty in many regions of the world, it is increasingly important to further explore drivers of declines in endangered species. Particularly, this framework can help management and modeling efforts by detecting changing levels of predictability at various timescales. In general, this framework could be extended to study nonlinear and multivariate interactions for different parts of salmon life cycles to further study carry-over effects, model predictive skill, and shifting drivers of ocean and freshwater survival.
